# Improvement of roughness in ultrasonic assisted magnetorheological finishing of small titanium alloy nuts by orthogonal test method

**DOI:** 10.1038/s41598-024-60153-z

**Published:** 2024-04-23

**Authors:** Cheng Bi, Axiang Ji, Hongyun Wang, Haibo Wang, Junhua Zhu, Fenfen Zhou

**Affiliations:** 1 School of Intelligent Manufacture, Taizhou University, Taizhou, 318000 Zhejiang China; 2 Technique Department, Zhejiang Feiya Elevator Co. Ltd, Jinhua, China

**Keywords:** Magnetorheological fluid (MR) finishing, Ultrasonic vibration, Surface roughness, Orthogonal tests, Titanium alloy, Mechanical engineering, Engineering, Materials science, Structural materials

## Abstract

Titanium alloy with high corrosion resistance, high strength-to-density ratio, and excellent biocompatibility has a wide range of applications in the field of biomedical implants. Polishing experiments of titanium alloy with a small size and complex shapes were investigated using an ultrasonic assisted magnetorheological finishing (UMRF) device excited by a three-pole magnetic field generator. The models of the normal force and the shear force were first proposed based on the Preston equation to analyze the mechanism of material removal in the UMRF process. Subsequently, the single-factor experiments using titanium alloy nuts (M3) and the MR polishing fluid with silicon carbide abrasives were carried out. Furthermore, to improve the surface roughness and the change rate of surface roughness of nuts, orthogonal tests with a standard L_9_(3^4^) orthogonal array were designed and performed based on the optimized process parameters obtained from the single-factor experiment. The results indicated the effect on surface roughness and change rate of surface roughness as applied current > roller speed > ultrasonic amplitude > spindle speed and applied current > roller speed > spindle speed > ultrasonic amplitude, respectively. Moreover, the surface roughness was improved from an initial 1.247 μm to a final 0.104 μm after the polishing for 80 min under these optimal process parameters.

## Introduction

Titanium alloy is an alloy composed of titanium and other elements, which has the advantages of a high strength-to-density ratio, high corrosion resistance, and excellent biocompatibility^1,2^. These excellent characteristics bring a bright application in modern industry, such as aircraft compressor discs and turbine blades, orthopedic implants, surgical instruments, and medical instruments^3,4^. Producing artificial screws/nuts with high performance using titanium alloy means acquiring good surface quality, which can reduce the entry of corrosion products into the body^5,6^. Furthermore, the requirements of a higher reliability and longer life for orthopedic implants can be met by obtaining a surface with lower damage and higher finish^7^. However, the artificial screws/nuts have the characteristics of small size and complex shape, which makes it difficult to obtain high accuracy. In addition, it is very difficult to obtain high-quality surfaces for titanium alloys due to the properties of the material itself of high hardness and poor thermal conductivity. Therefore, it is one of the technical problems to be solved urgently in ultra-precision polishing of artificial screw/nut.

Magnetorheological fluid (MR) is a smart material^8,9^, and it has been extensively used in MR dampers, MR finishing, MR clutches, etc.^10–13^. MR finishing, which uses the MR effect to form a "flexible polishing brush" on the surface of the polishing tool, is a typical flexible finishing technology^14–16^. It overcomes the shortcomings of easy pollution, low efficiency and low controllability of traditional electrochemical polishing^17,18^, magnetic abrasive finishing^19,20^, ultrasonic polishing technology^21,22^, and has advantages of high surface quality, low surface damage, high efficiency, low energy consumption, controllable material removal rate, etc.^23–25^. It has been successfully used in industrial production for ultra-precision machining of optical surfaces and semiconductor wafers^26,27^. For example, the surface roughness of optical glasses can reach Ra 0.86nm at a wheel speed of 1256 mm s^−1^ of wheel speed and magnetic field intensity of 15.92 kA m^−1^ by MR finishing^27^. This means that ultra-precision polishing of parts using MR fluids can be achieved by appropriate methods or devices.

According to the structure and magnetic field excitation principle, the existing MR finishing methods can be divided into two types. The MR finishing can be achieved by a "small grinding head" formed on the polishing wheel/ball by the MR polishing fluid having a local "spots" contact with the workpiece surface (the magnetic pole fixed inside)^24,26,27^, which is called the point-contact type. The MR finishing method based on the point-contact type is often used for polishing complex shape workpieces due to its small polishing tool. The polishing films can be formed on the polishing disc by the MR polishing fluid having a or a few area contacts with the workpiece surface (many small magnetic poles clustered)^15,28,29^, which is called area-contact type. This method based on the area-contact type is often used for polishing large-size workpieces due to the large polishing tool.

It can be seen that the common feature of the existing MR finishing is that a flexible polishing tool is formed on the surface of the polishing wheel/ball or disk under the coupling of the magnetic field and MR polishing fluid. The shortcomings of the existing MR finishing based on the point-contact or area-contact type is that the structure/shape/size of the polishing tool can not be adjusted. Therefore, for the workpieces with a small size and complex shapes, the polishing tools of existing MR finishing can not make effective contact with the workpiece surface due to their shape/size limitations. The MR finishing methods have become a technical bottleneck restricting its practical application, and its structural principle and polishing ability can not meet the needs of ultra-precision polishing of artificial screw/nut with small size and complex shape.

Different from the previous MR finishing methods, this paper presents an ultrasonic assisted magnetorheological finishing (UMRF) excited by a three-pole magnetic field generator, where the magnetic field can be extended into the tiny space by the brush wires with the permeability and the flexibility. The UMRF device consists of a magnetic field generator, an ultrasonic vibrator, a roller, some iron rods, and some brush wires. The MR polishing fluids are sealed in the roller, and are magnetized under the combined excitation of the magnetized brush wires and the applied magnetic field. Therefore, a lots of small brushes are formed on the surface of brush wires (called polishing brushes). It is easy for polishing brushes to reach the tiny space of workpieces. Moreover, polishing brushes can maintain a good fit with different curvature surfaces. Besides, it is beneficial for the workpiece to avoid or reduce being damaged due to the flexibility of polishing brushes.

Ultrasonic vibration technology with simple equipment, low cost, and strong versatility has been successfully applied in various industries for many years^21,30,31^. However, it has the shortcomings of uncontrollable material removal, low surface quality, and low efficiency. MR finishing technology has the advantages of high surface quality, controllable material removal, and high efficiency^32,33^, but it has certain requirements for the shape/size of the workpieces, and it is difficult to process the workpiece with small size and complex shape. Compared with the above polishing method, the innovation of this article is mainly manifested as following. First of all, the flexible polishing brushes will not produce compressive stress on the surface layer of the workpiece and they can reach tiny spaces, which can reduce or eliminate the dead end that can't be polished and obtain a high surface quality. Secondly, the hardness of the MR brushes can be controlled by adjusting the applied current, obtaining controllable material removal rates. Furthermore, a set of workpieces may be polished simultaneously by a set of polishing brush wires with the assistance of ultrasonic vibration, which can obtain high efficiency. Therefore, it is foreseeable that the UMRF method could help satisfy the ultra-precision polishing of artificial screw/nut. Moreover, this study will greatly promote the further development and practical application of MR finishing. During the research process, the mechanism of material removal of the UMRF method was first studied and analyzed. Subsequently, a UMRF device was fabricated and tested to verify the feasibility of the UMRF method. Finally, the influence of the different process parameters on the surface roughness and the material removal rate of the UMRF were examined by single-factor experiments and optimized by orthogonal experiments.

## Experiment

### Principle and setup

**Figure 1:**
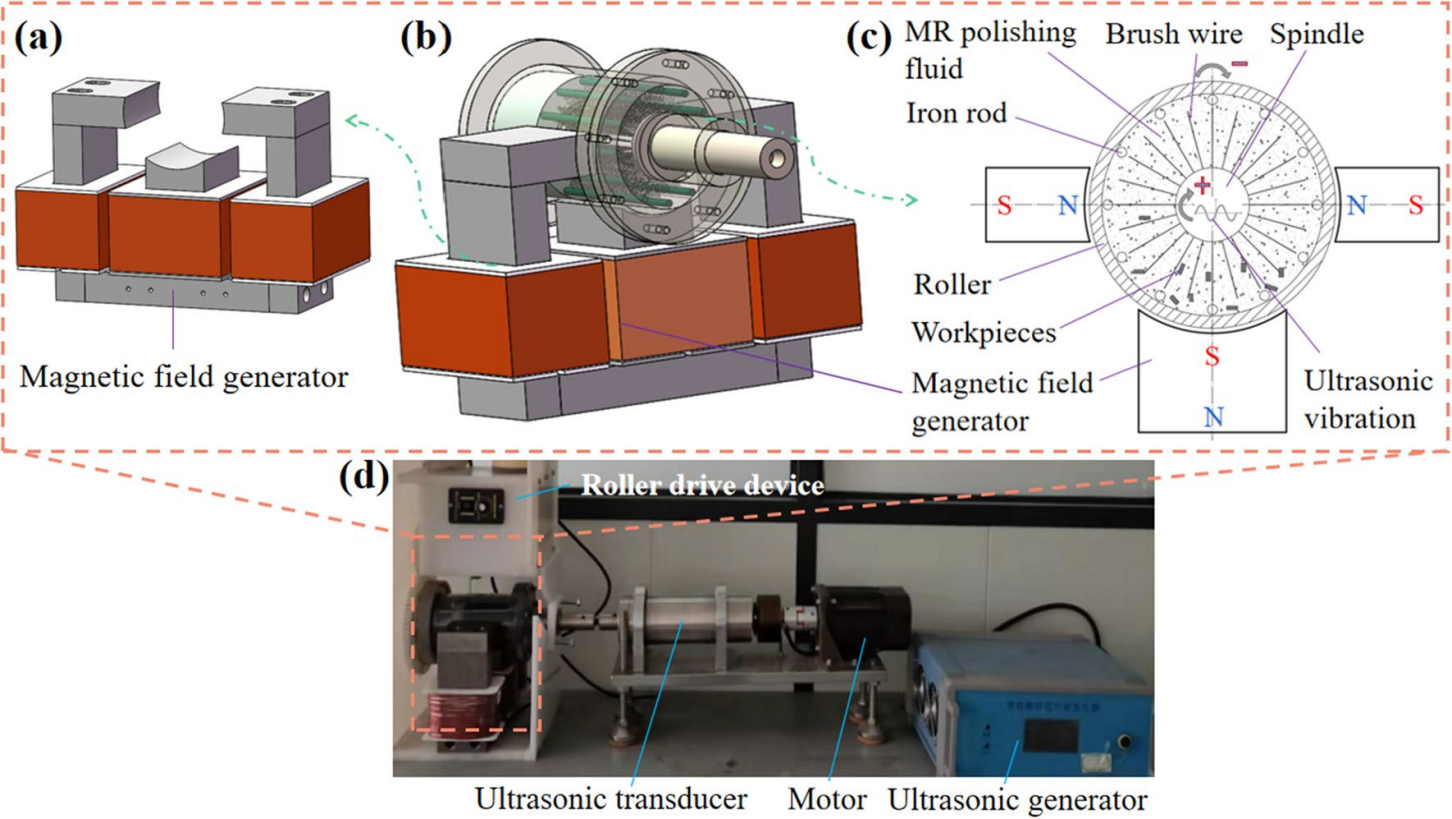
The schematic diagram of the experimental setup.

The principle and experimental setup in this paper is similar to that used in our former report^30^, as shown in Fig. 1. The main parts of the setup are composed of a spindle, brush wires, iron rods, a roller, a three-pole magnetic field generator, and a ultrasonic vibrator. The MR polishing fluids and the workpieces were placed between the spindle and the roller. The flexible and magnetic brush wires and the iron rod were fixed in the spindle and the roller, respectively. The spindle and the roller are driven by two motors to rotate oppositely. The brush wires and the MR polishing fluids will both be magnetized under the applied magnetic field, and the magnetized MR polishing fluids will form chain structures on the magnetized brush wires. If a proper relative movement between the roller and the spindle happens, the relative movement between the workpiece and the magnetic brush will be generated and the abrasives in the polishing brushes can remove the material on the workpiece surface. To generate enough magnetic field in the MR polishing fluid of polishing region, a three-pole magnetic field generator was designed. The copper wire with the diameter of 1.25 mm and the maximum input current of 5 A was wound in the pole with 1080 turns. The diameter of the roller was 90 mm. The iron core and the spindle were made of electromagnetic pure iron (DT4C) chosen due to high magnetic permeability and saturation magnetic induction.

### Mechanism of material removal

**Figure 2:**
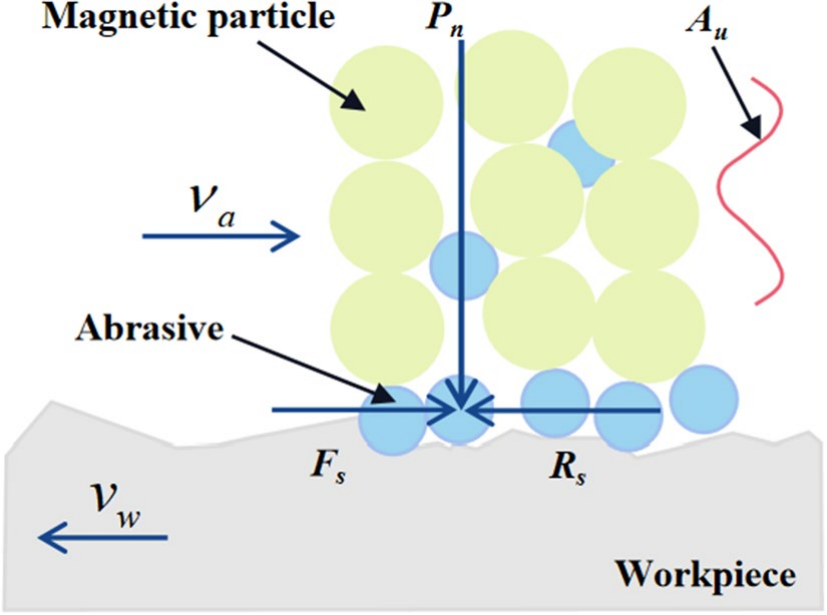
Schematic diagram of polishing process mechanism of UMRF.

In the UMRF method, polishing brushes, abrasives, and workpieces are in contact with each other. According to Preston equation^34,35^, the workpiece surface is mainly subjected to normal force *F*_*n*_ and shear force *F*_*s*_ under the action of magnetic chains when the relative movement between the workpiece and the magnetic brush happens, as shown in Fig. 2. *F*_*n*_ can cause the abrasives to indent on the surface of the workpiece. *F*_*s*_ mainly plays a role in the micro-cutting on the workpiece surface. *R*_s_ is the friction force. The stronger the magnetic field, the higher the shear resistance of the magnetic chains will be. Therefore, the cutting ability of abrasives is mainly determined by the depth at which the abrasive particles are pressed by *F*_*n*_. If *F*_s_ is higher than *R*_s_, the abrasives will move and the peaks of surface will be removed. In addition, When the shear force *F*_*s*_ is less than the friction force *R*_*s*_, the abrasives will roll or slip relative tothe workpiece surface. The normal force *P*_*n*_ (N/m^2^) can be mainly expressed by the magnetic field pressure *P*_*m*_ (N/m^2^) and the impact pressure *P*_*u*_ (N/m^2^) as Ref.^36^

1 $$P_{n} = P_{m} + P_{u}$$

To formulate a microstructure based constitutive model of the MR polishing fluid and to avoid complexity, the following assumptions are made:

- All the particles of carbon iron powders and abrasives are made of the same spheres with identical diameter and uniformly dispersed in the MR polishing fluid.
- The magnetic field produced by the magnetized carbonyl iron powder is not considered.
- Magnetic leakage and magnetic loss are not considered.
- The properties of MR polishing fluid do not change during polishing.

When the direction of the applied magnetic field is perpendicular to the workpiece surface, the pressure generated by a gradient magnetic field acting on the workpiece surface can be expressed as

2 $$P_{m} = \mu_{0} \int_{0}^{H} {M_{f} dH}$$

where *μ*_*0*_ is the vacuum permeability (H/m), *M*_*f*_ is the magnetization intensity of fluid, and *H* is the intensity of magnetic field (A/m).

According to the theory of field-induced polarization^23,38^, the magnetic moment of a magnetized dipolar particle *m* under the applied magnetic field can be described as

3 $$m = \frac{4}{3}\pi r^{3} \chi H$$

where *χ* is the susceptibility, and *r* is the radius of the particle (m).

If the magnetization intensity of magnetic particle in MR polishing fluid is *M* and the particle volume fraction of fluid is*φ*, the magnetization intensity of MR polishing fluid *M*_*f*_ can be expressed as^37,38^

4 $$M_{f} = \varphi \chi H$$

Combining Eqs. (2) and (4), *P*_*m*_ can be expressed as

5 $$P_{m} = \varphi \chi \int_{0}^{H} {HdH}$$

Considering an angle *β*_*1*_ (0° ≤ *β*_*1*_ ≤ 90°) between the magnetic field direction and the workpiece surface due to the irregular movement of workpiece in the roller, the magnetic field force *P*_*mf*_ will become

6 $$P_{mf} = P_{m} \sin \beta_{1} = \varphi \chi \sin \beta_{1} \int_{0}^{H} {HdH}$$

The abrasives clamped by magnetic particles continuously impact the workpiece surface under the action of ultrasonic waves. The contact surface between the abrasive and the workpiece can be seen as an infinite elastic surface because the abrasive diameter is in the order of microns.

According to the Hertz contact elastic contact theory, when the abrasives strike the workpiece surface vertically, the impact pressure *P*_*u*_ can be expressed as

7 $$P_{u} = \frac{1}{3}\left( {\frac{5\pi }{4}\rho } \right)^{\frac{3}{5}} \left( {\frac{{1 - v_{1}^{2} }}{{E_{1} }} + \frac{{1 - v_{2}^{2} }}{{E_{2} }}} \right)^{{ - \frac{2}{5}}} D^{2} \left( {\frac{{4f\rho_{0} V_{0} A_{u} e^{{ - \lambda h_{x} }} }}{{V_{1} }}} \right)^{\frac{6}{5}}$$

where *v*_*1*_ and* v*_*2*_ is the Poisson's ratio of abrasive and workpiece, respectively; *E*_1_ (N/m^2^) and *E*_2_ (N/m^2^) is the elastic modulus of abrasive and workpiece, respectively; *ρ* (kg/m^3^) and *V*_*n*_ (m/s) is the density and the impact speed of abrasive, respectively; D is the diameter of the particle (m). The propagation velocity of ultrasonic wave in MR polishing fluid under the applied magnetic field is attenuated. Assuming that the attenuation coefficient is *λ* and the initial sound pressure is *p*_*0*_ (N/m^2^), the impact speed *V*_*n*_ of the abrasive can be described as

8 $$V_{n} = \frac{{p_{0} e^{{ - \lambda h_{x} }} }}{{\rho V_{1} }}$$

where *h*_*x*_ is the distance between the workpiece and the adjacent brush (m), and *V*_*1*_ is the propagation speed of ultrasonic wave in the MR polishing fluid (m/s).

According to the ultrasonic principle, the initial sound pressure *p*_*0*_ can be described as

9 $$p_{0} = 4f\rho_{0} V_{0} A_{u}$$

where *f* is the frequency of the ultrasonic vibration (Hz), *ρ*_0_ is the material density of the polishing brush (kg/m^3^), and *A*_*u*_ is the ultrasonic amplitude (m).

Combining Eqs. (7), (8), and (9), we arrive at

10 $$P_{u} = KD^{2} \left( {\frac{{4f\rho_{0} V_{0} A_{u} e^{{ - \lambda h_{x} }} }}{{V_{1} }}} \right)^{\frac{6}{5}}$$

where K is parameters defined by

$${\rm K} = \left( {\frac{5\pi }{4}\rho } \right)^{\frac{3}{5}} \left( {\frac{{1 - v_{1}^{2} }}{{E_{1} }} + \frac{{1 - v_{2}^{2} }}{{E_{2} }}} \right)^{{ - \frac{2}{5}}}$$

**Figure 3:**
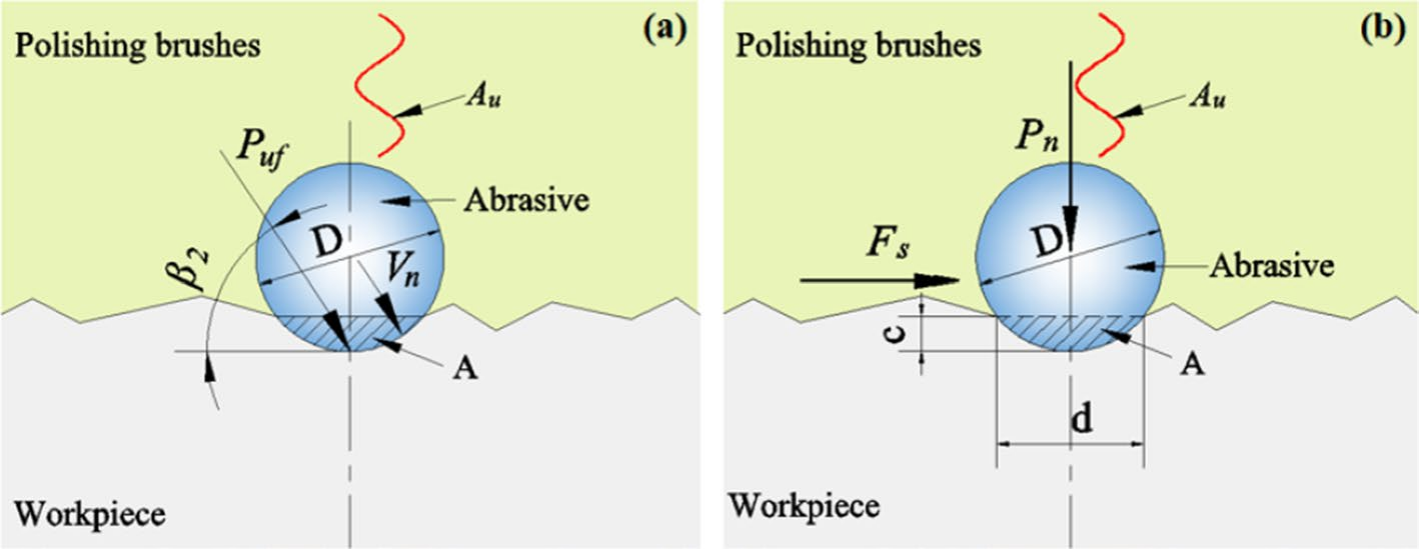
Geometry of contact of (**a**) the impact pressure; (**b**) normal force between abrasive and workpiece for UMRF.

Similarly, considering the impact direction of the abrasives is not perpendicular to the surface of the workpiece and assuming their angle *β*_*2*_ (0° ≤ *β*_*2*_ ≤ 90°), as shown in Fig. 3a, the impact pressure will become

11 $$P^{\prime}_{u} = P_{u} \sin \beta_{2} = KD^{2} \left( {\frac{{4f\rho_{0} V_{0} A_{u} e^{{ - \lambda h_{x} }} }}{{V_{1} }}} \right)^{\frac{6}{5}} \sin \beta_{2}$$

Combining Eqs. (1), (6), and (11), the total normal force *P*_*n*_ exerted by the abrasive on the workpiece surface can be rewritten as

12 $$P_{n} = P^{\prime}_{m} + P^{\prime}_{u} = \varphi \chi \sin \beta_{1} \int_{0}^{H} {HdH} + KD^{2} \left( {\frac{{4f\rho_{0} V_{0} A_{u} e^{{ - \lambda h_{x} }} }}{{V_{1} }}} \right)^{\frac{6}{5}} \sin \beta_{2}$$

In the UMRF method, the abrasives will produce the certain indentation on the surface of workpiece under the action of positive pressure, and the central cross-section of the indentation is consistent with the outline of the abrasive particles pressed into the surface. Figure 3b shows the contact cross-section between the workpiece and the abrasive in the polishing brush. The depth and the width of indentation is defined as* c* (m) and* d* (m), respectively. The contact area is defined as *A* (m^2^). According to the Hertz contact of spherical surface, the indentation width *d* can be expressed as

13 $$d = \sqrt {D^{2} - \left( {\frac{{\pi D^{2} H_{BHN} - 2P_{n} }}{{\pi DH_{BHN} }}} \right)}$$

where $H_{BHN} = {{2P_{n} } \mathord{\left/ {\vphantom {{2P_{n} } {\pi D\left( {D - \sqrt {D^{2} - d^{2} } } \right)}}} \right. \kern-0pt} {\pi D\left( {D - \sqrt {D^{2} - d^{2} } } \right)}}$ is the Brinell hardness value of the workpiece.

The indentation depth c can be calculated as

14 $$c \approx \frac{D}{2} - \frac{1}{2}\sqrt {D^{2} - d^{2} }$$

The contact area A can be expressed as

15 $$A = \frac{{D^{2} }}{4}\sin^{ - 1} \left[ {2\sqrt {\frac{c}{D}\left( {1 - \frac{c}{D}} \right)} } \right] - \frac{1}{2}\sqrt {c\left( {D - c} \right)} \cdot \left( {D - 2c} \right)$$

Therefore, the shear force *F*_*s*_ can be expressed as

16 $$F_{s} = A \cdot \tau + P_{m} \cdot \cos \beta_{1} + P_{u} \cdot \cos \beta_{2}$$

where *τ* is the shear stress of polishing MR fluid (N/m^2^), which can be measured by the MR rheometer.

Combining Eqs. (5), (10), (15), and (16), we arrive at

17 $$\begin{gathered} F_{s} = \tau \cdot \left\{ {\frac{{D^{2} }}{4}\sin^{ - 1} \left[ {2\sqrt {\frac{c}{D}\left( {1 - \frac{c}{D}} \right)} } \right] - \frac{1}{2}\sqrt {c\left( {D - c} \right)} \cdot \left( {D - 2c} \right)} \right\} \hfill \\ \quad \;\;\; + \varphi \chi \cos \beta_{1} \int_{0}^{H} {HdH} + KD^{2} \left( {\frac{{4f\rho_{0} V_{0} A_{u} e^{{ - \lambda h_{x} }} }}{{V_{1} }}} \right)^{\frac{6}{5}} \cos \beta_{2} \hfill \\ \end{gathered}$$

Equation (18) shows that *F*_*s*_ is mainly determined by the shear stress *τ* of MR polishing fluid, properties of MR polishing fluids, magnetic field strength *H*, the propagation speed of ultrasonic wave in the MR polishing fluid *V*_*1*_, and the ultrasonic amplitude *A*_*u*_. Therefore, the MR polishing fluid properties (such as *τ*,*φ*, D), the enameled wire specification, the number of turns, the value of applied current *I* and ultrasonic amplitude *A*_*u*_, spindle speeds *n*_*1*_, roller speeds *n*_*2*_ can be designed and determined according to Eq. (17).

### Materials

The polished workpieces of titanium alloy nuts (M3) were obtained from Taizhou Lihong Stainless Steel Products Co., Ltd. (China). Carbon iron powders (CIPs), which are the magnetic particles, with an average particle size of 3 μm were supplied by Guangzhou Metal Materials Co., Ltd. (China). And silicon carbides (SiCs), which are abrasives, with an average particle size of 4 μm were provided from Shanghai Metal Powder Scientific Research Co., Ltd. (China).

### Design of experiments

**Figure 4:**
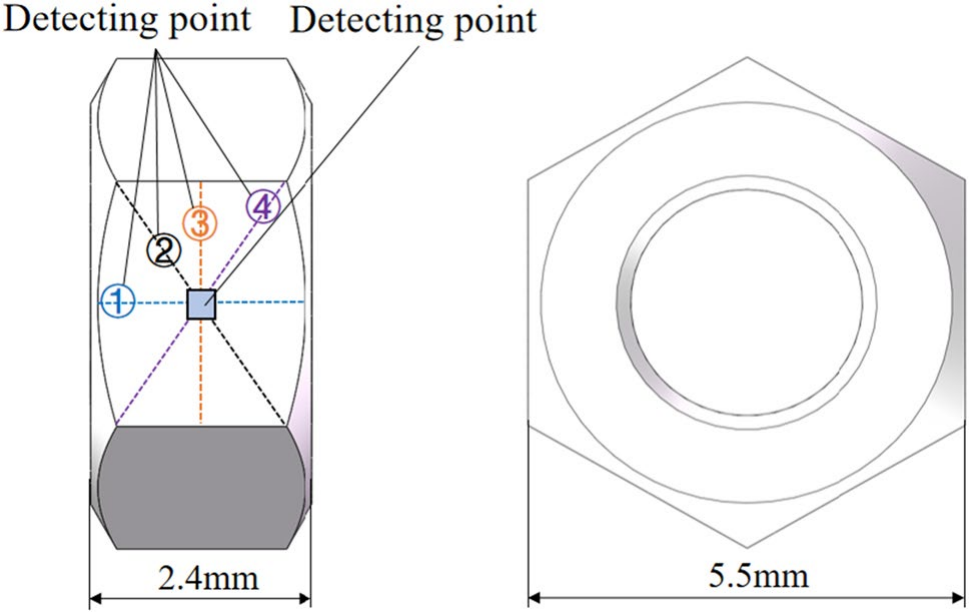
Diagram of detection point and line distribution of workpiece surface.

For each polished titanium alloy nut, the surface roughness of four-point measured method was adopted, as shown in Fig. 4. There are four detection lines, which are respectively the center line and diagonal line of the workpiece surface to be detected. The surface roughness value obtained by the experiment is the average value of the measured surface roughness under the four detection lines. The process parameters have a great influence on the surface quality of the workpiece^30,32^. The surface topography inspection point was located in the center of the surface of the measured workpiece and the size of the acquisition point is 0.381 mm × 0.381 mm. The total magnification of the objective lens was 700 times and the observation was made in a bright field environment. Therefore, the effects of the four parameters, i.e., spindle speed *n*_*1*_, the roller speed *n*_*2*_, the ultrasonic amplitude *A*_*u*_, and the applied current *I* on the nut’s surface roughness and the change rate of surface roughness were studied by the orthogonal experimental method.

The first set of experiments was designed based on the single-factor experiment. Effects of spindle speed *n*_*1*_ at the different roller speeds *n*_*2*_ (50, 70, 90, and 110 r/min), ultrasonic amplitudes *A*_*u*_ (0, 5, 10 and 15 μm), and applied currents *I* (2, 3, 4, and 5 A) on finishing performance have been parametrically studied.

The second set of experiments was designed based on the orthogonal experiment. In the standard L_9_(3^4^) orthogonal array, four factors A, B, C, and D were defined as spindle speeds *n*_*1*_, roller speeds *n*_*2*_, ultrasonic amplitudes *A*_*u*_, and applied currents *I*, respectively. Each factor in the array had three levels. Therefore, according to different combinations of factors and their levels, nine test experiments were designed. As well as the combination of parameters was also optimized.

If the tested surface roughness is *Ra*, the change rate of surface roughness △*Ra* can be represented as

18 $$\Delta Ra = \frac{{Ra_{1} - Ra_{2} }}{{Ra_{1} }} \times 100\%$$

where *Ra*_*1*_ is the surface roughness before finishing, *Ra*_*2*_ is the surface roughness after finishing.

## Results and discussion

### Morphology and structure

**Figure 5:**
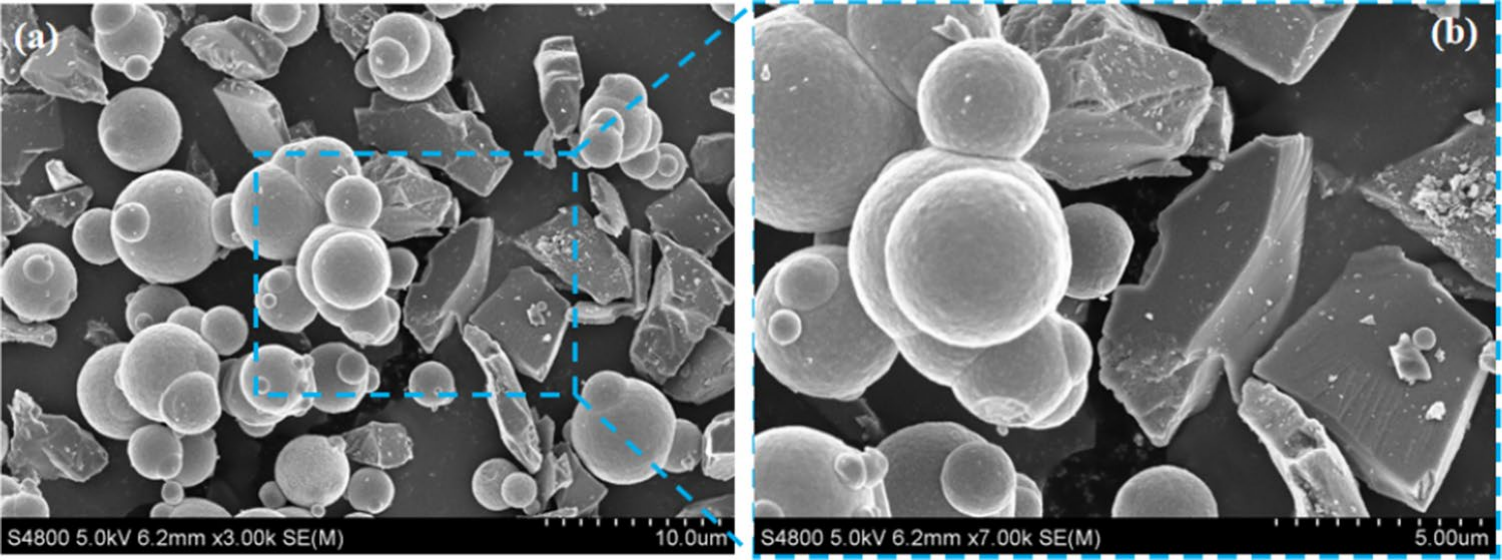
SEM images of CIP and SiC mixed together.

The MR polishing fluids were composed of silicone oil (38 wt%), CIPs (35 wt%), SiCs (23 wt%), and additives (C_18_H_35_NaO_2_, OP-10, 4 wt%). The morphology of CIP and SiC mixed were shown in Fig. 5a. Figure 5b was taken at 7000 magnification. It shows clearly that the CIP particles were nearly spherical with an average particles size of approximately 3 µm, whereas SiC particles were polygon in shape with sharp edges and an average particles size of about 4 μm. The sharp edges were more useful for cutting the workpiece.

### Single-factor experiment

**Figure 6:**
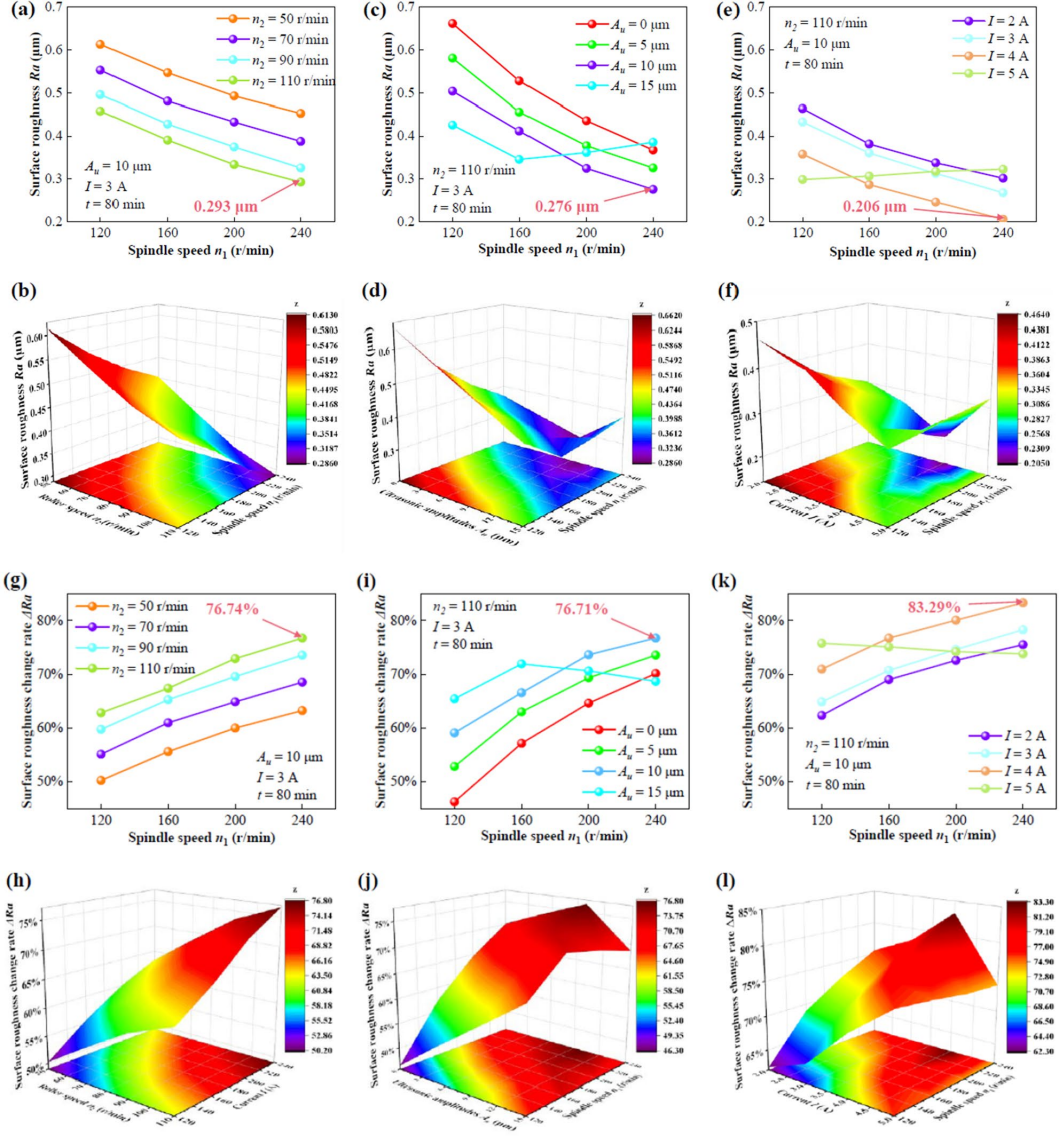
The *Ra* and △*Ra* versus *n*_*1*_ under different *n*_*2*_ (**a**,**b**,**g**,**h**); *A*_*u*_ (**c**,**d**,**i**,**j**); *I* (**e**,**f**,**k**,**l**), respectively.

Single-factor experiments were designed to explore the influence of various process parameters on the surface roughness and the change rates of surface roughness of the workpiece. The curves of surface roughness *Ra* and the change rates of surface roughness △*Ra* at different spindle speeds *n*_*1*_, roller speeds *n*_*2*_, ultrasonic amplitudes *A*_*u*_, and applied currents *I* were shown in Fig. 6. As shown in Fig. 6a,b, *Ra* exhibited a decreasing trend with the increase of *n*_*2*_ as the spindle speeds *n*_*1*_ gradually increased under *A*_*u*_ = 10 μm, *I* = 3 A, and *t* = 80 min. In detail, the surface roughness showed a *Ra*_*min*_ of 0.293 μm at *n*_*1*_ = 240 r/min with a *n*_*2*_ of 110 r/min. *Ra* showed a decreasing trend with the increase of *A*_*u*_ under *I* = 3 A, *n*_*2*_ = 110 r/min, and *t* = 80 min except for *A*_*u*_ = 15 μm, as shown in Fig. 6c,d. It indicates an improved *Ra*_*min*_ value of 0.276 μm at *n*_*1*_ = 240 r/min and *A*_*u*_ = 10 μm. Figure 6e,f also presented a decreasing trend of *Ra* with the increase of applied current under *A*_*u*_ = 10 μm, *n*_*2*_ = 110 r/min, and *t* = 80 min except for *I* = 5 A. While an improved *Ra*_*min*_ value of 0.206 μm happened at *n*_*1*_ = 240 r/min with a applied current of 4 A, which was superior to that with a applied current of 3 A, compared with Fig. 6a,b.

The corresponding change rates of surface roughness △*Ra* were shown in Fig. 6g,h. It shows The trends of △*Ra* are the opposite of those of *Ra*. △*Ra* steadily increased with the increase of the spindle speed *n*_*1*_ at different roller speeds *n*_*2*_, as shown in Fig. 6g,h. When *n*_*2*_ was 110 r/min, △*Ra* increased from 62.85 to 76.72% with the increase of *n*_*1*_ from 120 r/min to 240 r/min. △*Ra* exhibited a similarly increasing trend at different *A*_*u*_ and *I*, except for *A*_*u*_ = 15 μm and *I* = 5 A, respectively, as shown in Fig. 6i,j. The change rates of surface roughness showed a △*Ra*_*max*_ of 76.72% at *n*_*1*_ = 240 r/min with *A*_*u*_ of 10 μm. An improved △*Ra*_*max*_ value of 83.29% at *n*_*1*_ = 240 r/min with an applied current of 4 A. However, when *A*_*u*_ = 15 μm, △*Ra* increased from 65.45% to 71.93% in the spindle speed *n*_*1*_ from 120 r/min to 160 r/min, then decreased to 68.69% with the further increase in *n*_*1*_ to 240 r/min, as shown in Fig. 6i,j. When *I* = 5 A, △*Ra* slightly decreased from 75.75% to 73.79% in the spindle speed *n*_*1*_ from 120 r/min to 240 r/min, as shown in Fig. 6k,l.

These results indicate that the initial polishing process parameter combination in the single-factor experiments could be obtained as *n*_*1*_ = 240 r/min, *n*_*2*_ = 110 r/min, *A*_*u*_ = 10 μm, and *I* = 4A.

Generally, the surface before finishing is relatively rough with the convex peaks and burrs. The increasing of *n*_*2*_ inevitably leads to enlarging the relative speed between polishing abrasive and workpiece, resulting in the increase of the number of contacts between polishing abrasive and workpiece. Thus, the greater *n*_*2*_ will lead to a lower *Ra* and a higher △*Ra*, as shown in Fig. 6a,g. The material removal process of MR polishing is mostly completed by shearing behavior of the abrasive on the workpiece surface. However, the shear force of abrasives can be enhanced by the surface of the workpiece is impacted by ultrasonic vibration, which results in a lower *Ra* and a higher *η*, as shown in Fig. 6c,i. If *A*_*u*_ is too great, the impact pressure will deepen the indentation depth of the abrasives on the workpiece surface. As a consequence, new scratches or pits will be generated on the surface of the workpiece, resulting in a higher *Ra* and a lower *η*. As everyone knows, the shear force of MR fluid increases with the increase of the applied magnetic field or current. Therefore, the magnitude of clamping force of magnetic particles on abrasives mainly depends on the magnitude of the applied current. The higher the applied current, the higher the shear force of abrasives, and the more effectively the material can be removed, as shown in Fig. 6e,k. Similar to the effect of *A*_*u*_ on *Ra*, the extreme current leads to the excessive shear force, resulting in the new scratches on the surface of workpiece. As a consequence, this generates a slightly increase in *Ra.*

### Orthogonal experiment

#### Tests design and results

According to the single-factor experimental results, a set of initial optimized polishing process parameters were obtained. Based on the orthogonal analysis method^39^, a L_9_(3^4^) orthogonal array was designed by selecting the initial optimized parameters, as listed in Table 1. In this array, four factors A, B, C, and D were defined as spindle speeds *n*_*1*_, roller speeds *n*_*2*_, ultrasonic amplitudes *A*_*u*_, and applied currents *I*, respectively. Each factor in the array had three levels. The orthogonal test method can analyze the combined effects of multiple factors with fewer tests^39^. Therefore, the total nine experiments were designed and tested according to different combinations of factors and their levels. The orthogonal experimental results of workpiece final *Ra* and Δ*Ra* in different test runs for four factors were also summarized in Table 1. The average initial *Ra* of Titanium alloy nuts was about 1.233 μm.

**Table 1:** Test runs design based on orthogonal array and their results.

Test No	Combination	Factor A *n*_*1*_ (r/min)	Factor B *n*_*2*_ (r/min)	Factor C *A*_*u*_ (μm)	Factor D *I* (A)	Average final *Ra* (μm)	Average Δ*Ra* (%)
1	A_1_B_1_C_1_D_1_	240	100	8	3.5	0.158	86.74
2	A_1_B_2_C_2_D_2_	240	120	10	4	0.108	90.83
3	A_1_B_3_C_3_D_3_	240	140	12	4.5	0.169	85.27
4	A_2_B_1_C_2_D_3_	260	100	10	4.5	0.171	83.46
5	A_2_B_2_C_3_D_1_	260	120	12	3.5	0.112	89.67
6	A_2_B_3_C_1_D_2_	260	140	8	4	0.108	90.13
7	A_3_B_1_C_3_D_2_	280	100	12	4	0.117	89.29
8	A_3_B_2_C_1_D_3_	280	120	8	4.5	0.172	87.42
9	A_3_B_3_C_2_D_1_	280	140	10	3.5	0.105	90.81

The results of the orthogonal tests are shown in Table 2, in which *K* and *G* represents the means of the surface roughness *Ra* after polished measured below the same level of the column parameters and that of the corresponding change rate of surface roughness Δ*Ra*, respectively. *R* represents the range, which is used to characterize the influence degree of each factor on the respective indices. According to Table 2, *R* was taken as the index.

**Table 2:** Orthogonal test analysis and results of *Ra* and Δ*Ra*.

Indexes		A (*n*_*1*_)	B (*n*_*2*_)	C (*A*_*u*_)	D (*I*)
*Ra*	*K*_*1*_	0.145	0.149	0.146	0.125
	*K*_*2*_	0.130	0.131	0.128	0.111
	*K*_*3*_	0.131	0.127	0.133	0.171
	*R*	0.015	0.021	0.018	0.06
	Factor influence sequence	D > B > C > A			
Δ*Ra*	*G*_*1*_	87.61	86.50	88.10	89.07
	*G*_*2*_	87.75	89.31	88.37	90.08
	*G*_*3*_	89.17	88.74	88.08	85.38
	*R*	1.56	2.81	0.29	4.7
	Factor influence sequence	D > B > A > C			

#### Analysis of level average response

**Figure 7:**
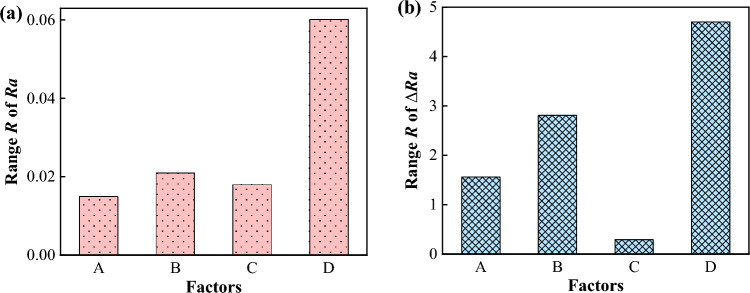
Range of *Ra* (**a**) and Δ*Ra* (**b**) for factors of A (*n*_*1*_), B (*n*_*2*_), C (*A*_*u*_), and D (*I*).

The range values of four factors in the orthogonal experiment are shown in Fig. 7. For *Ra*, Fig. 7a shows that the range of applied current was the largest, followed by the roller speed and the spindle speed, and the ultrasonic amplitude was the smallest. Therefore, it could be deduced that the influence of each factor on the *Ra* is *I* (0.06) > *n*_*2*_ (0.021) > *A*_*u*_ (0.018) > *n*_*1*_ (0.015). For the change rate of surface roughness Δ*Ra*, Fig. 7b depicts that the range of applied current was still the largest, followed by the roller speed. However, the next order was the spindle speed and the ultrasonic amplitude, which was different from that on *Ra*. Therefore, it could be deduced that the range *R* of each factor on Δ*Ra* could be expressed as *I* (4.7) > *n*_*2*_ (2.81) > *n*_*1*_ (1.56) > *A*_*u*_ (0.29). In brief, it is found that the applied current and the roller speed both significantly had the greater effects on *Ra* and Δ*Ra*, the spindle speed and the ultrasonic amplitude had a less effect.

#### Analysis of level average response

**Figure 8:**
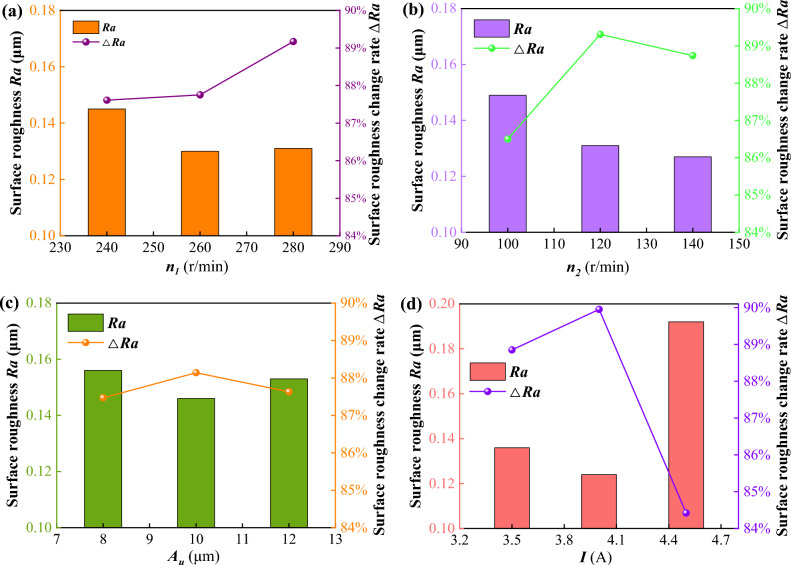
The effects of four parameters of level mean response on *Ra* and Δ*Ra.*

The analysis of level mean response is commonly used to examine the influence of various factors on the target at different levels to find the optimal combination of conditions to achieve the best results^40^. In this orthogonal experiment, the effects of four parameters of level mean response on *Ra* and Δ*Ra* were illustrated in Fig. 8.

Figure 8a indicates that the spindle speed had great influences both on *Ra* and Δ*Ra*. *Ra* and Δ*Ra* were both promoted with the spindle speed increasing. It seems that the higher value of spindle speed should be better. This is mainly because the high spindle speed led to more contact between the workpiece surface and the abrasive. Moreover, if the spindle speed was too great, the larger micro-cutting depth would be achieved by the abrasives caused by significant centrifugal force. However, the stability of the entire experimental polishing device became worse under *n*_*1*_ = 280 r/min. Therefore, it is appropriate in the present paper to choose the value of *n*_*1*_ = 260 r/min.

Figure 8b indicates that the roller speed significantly contributed to the decrease of *Ra* and increase of Δ*Ra* under *n*_*2*_ = 120 r/min. Differently, *Ra* was continuously improved with the increase of roller speed, however, Δ*Ra* became worse under *n*_*2*_ = 140 r/min. This might be due to the relative speed between the polishing brush and the workpiece. This relative speed would certainly be enlarged due to the increase in roller speed. Generally, the greater relative speed would result in the higher material removal. However, the total number of abrasives in the MR polishing fluid decreased in the UMRF because some of them were thrown to the outer edge of the roller by centrifugal force under the higher roller speed. Therefore, the value of roller speed should be chosen not too high or not too low. It is appropriate in the present paper to choose the value of *n*_*2*_ = 120 r/min.

Figure 8c indicates that the ultrasonic amplitude had a great influence both on *Ra* and Δ*Ra* under *A*_*u*_ = 10 μm. However, *Ra* and Δ*Ra* did not significantly contribute to the increase but got worse with the increasing *A*_*u*_. This was probably because the large ultrasonic vibration could lead to the high shear and friction force of abrasives on the peak and valley of workpiece surface. But the larger the ultrasonic vibration was applied, the deeper the micro-cutting depth and the worse the surface roundness due to the new scratches or pits. Therefore, it is appropriate in the present paper to choose the value of *A*_*u*_ = 10 μm, which agrees with the other results^40^.

Figure 8d indicates that the applied current had a large influence on improving both *Ra* and Δ*Ra* under *I* = 4 A while worse under the applied current of 5 A. This might be due to the high applied current leading to the great clamping force of magnetic particles on abrasives. Generally, the higher the current was applied, the greater the shear force was achieved on the polishing brush. However, if the applied current was too high, excessive shear force would be produced, which could lead to a deeper micro-cutting depth on the workpiece surface. Therefore, it is appropriate in the present paper to choose the value of *I* = 4 A.

Summarizing the above, to achieve the good *Ra* and the high Δ*Ra*, the optimal combination of process parameters was selected as *n*_*1*_ = 240 r/min, *n*_*2*_ = 120 r/min, *A*_*u*_ = 10 μm, and *I* = 4 A in this study.

**Figure 9:**
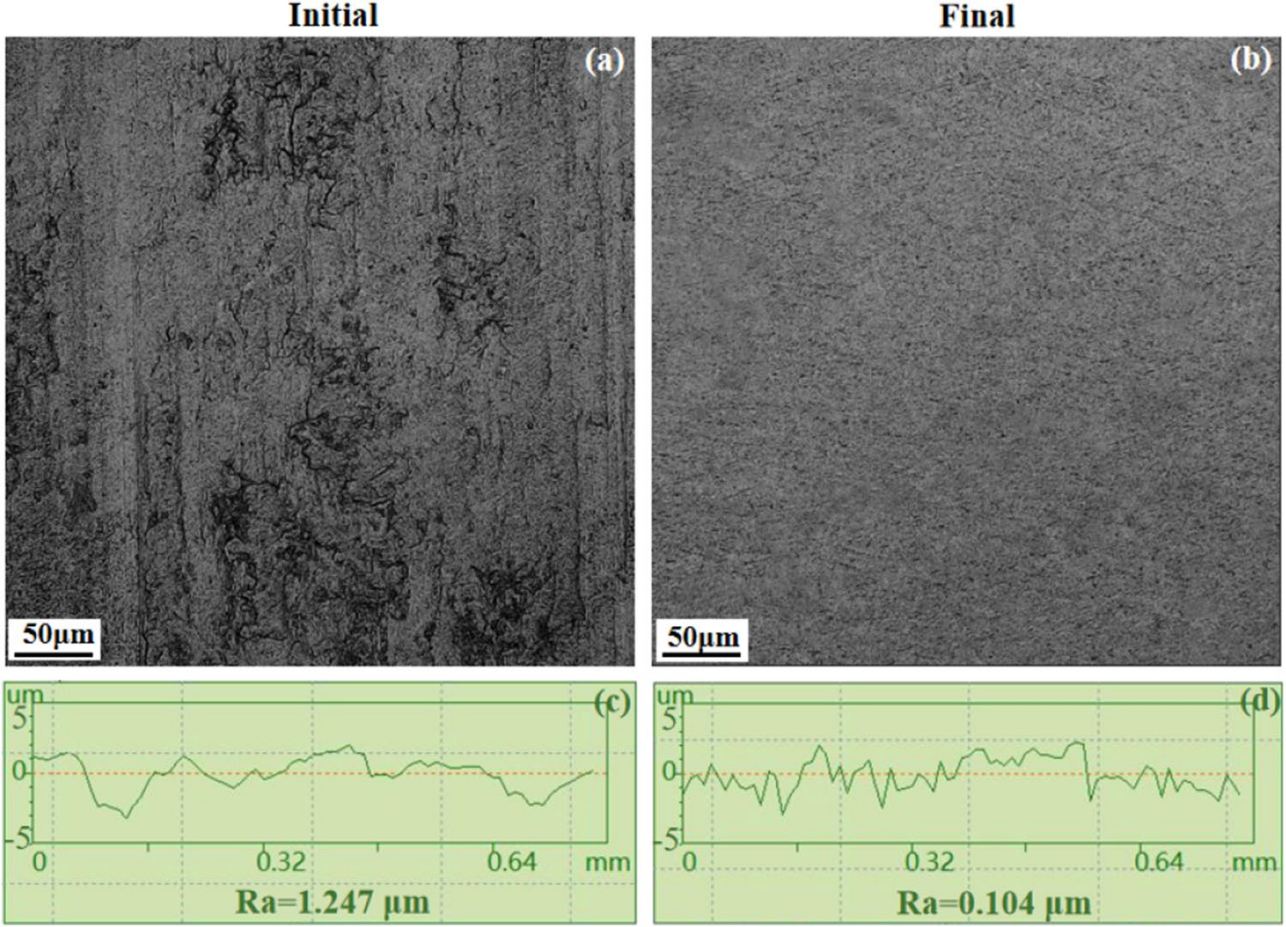
Comparison of surface morphology and measured surface roughness before polishing (**a**) and (**c**), and after polishing (**a**) and (**c**), respectively.

**Figure 10:**
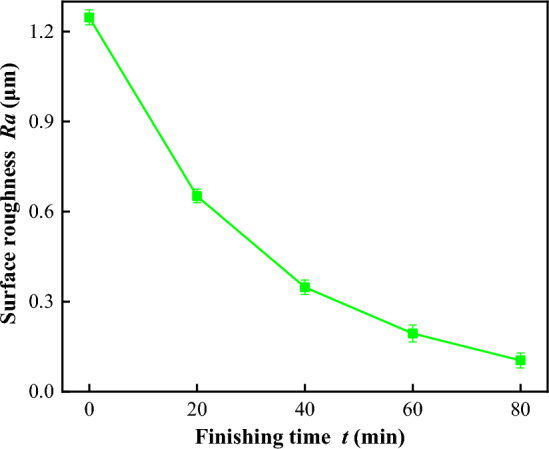
*Ra* versus *t* for optimal process conditions and control experiments.

The finishing experiments of UMRF were performed on titanium alloy nuts under the optimal process parameters. The workpieces were machined four times for a total of 80 min. The typical surface morphologies and the surface roughness, which were characterized using an ultra-depth-of-field microscope (Olympus DSX1000, Japan) and surface roughometer (CH80, China), were shown in Fig. 9. Figure 9a,b show that the initial surface morphology with amount of micro/nano peak-valley structures was greatly and effectively improved under optimal process parameters, although there are still relatively shallow/less valleys in the final surface. The value of *Ra* was decreased from an initial 1.247 μm to a final 0.104 μm, as shown in Fig. 9c,d, respectively. A measured result of *Ra* under the optimal process parameters of *n*_*1*_ = 240 r/min, *n*_*2*_ = 120 r/min, *A*_*u*_ = 10 μm, and *I* = 4 A. is shown in Fig. 10.

## Conclusion

In this study, an ultrasonic assisted magnetorheological finishing (UMRF) device was used with a custom three-pole excitation electromagnet to polish the titanium alloys with a small size and complex shapes. The force model was established based on the Preston equation to analyze the mechanism of material removal in the UMRF process. A series of single-factor experiments were designed and performed on titanium alloy nuts (M3). The results showed that the initial optimal polishing process parameter combination were *n*_*1*_ = 240 r/min, *n*_*2*_ = 110 r/min, *A*_*u*_ = 10 μm, and *I* = 4A. Further, orthogonal experiments were designed and performed based on the parameters obtained by the single-factor experiments. The results indicated the effect on *Ra* and Δ*Ra* as *I* > *n*_*1*_ > *A*_*u*_ > *n*_*2*_ and *I* > *n*_*1*_ > *n*_*2*_ > *A*_*u*_, respectively. Under these optimal process parameters of *n*_*1*_ = 240 r/min, *n*_*2*_ = 120 r/min, *A*_*u*_ = 10 μm, and *I* = 4A, the surface roughness was reduced from 1.247 μm to 0.104 μm after the polishing for 80 min. It is foreseeable that UMRF is a highly adaptable and efficient method for polishing titanium alloy with a small size and complex shapes.

## Data Availability

All data generated or analyzed during this study are included in this manuscript.
